# Involvement of D1- and D2-like dopamine receptors in the hippocampal CA1 region in mediating the restraint stress-induced analgesia in the rats

**DOI:** 10.1016/j.ibneur.2026.02.016

**Published:** 2026-02-21

**Authors:** Diba Shirmohammadi, Homayoon Golmohammadi, Shima Abtin, Abbas Haghparast

**Affiliations:** a School of Medicine, Iran University of Medical Sciences, Tehran, Iran; b Neurobiology Research Center, Institute of Neuroscience and Cognition, Shahid Beheshti University of Medical Sciences, Tehran, Iran; c Neuroscience Research Center, Institute of Neuroscience and Cognition, Shahid Beheshti University of Medical Sciences, Tehran, Iran; d School of Cognitive Sciences, Institute for Research in Fundamental Sciences, Iran; e Department of Basic Sciences, Iranian Academy of Medical Sciences, Tehran, Iran; f National Brain Mapping Laboratory (NBML), Tehran, Iran

**Keywords:** Acute pain, Stress, D1-like dopamine receptor, D2-like dopamine receptor, CA1 region of hippocampus, Tail-flick test, Restraint stress, Rat

## Abstract

Acute stress exposure can elicit analgesic responses in multiple pain models. Research indicates that dopaminergic pathways in the hippocampus contribute to the analgesic responses elicited by forced swim stress, considered a combination of physical and psychological stress. Since psychological and physical stresses can trigger different brain pathways and areas, this study has examined how dopaminergic receptors in the CA1 region of the hippocampus affect the pain relief induced by restraint stress (RS), generally considered a psychological stress, in an acute pain model. Ninety-six Wistar rats underwent stereotaxic surgery, during which a cannula was unilaterally implanted in the CA1 region of the hippocampus. Five minutes prior to exposure to RS, D1- and D2-like dopamine receptor antagonists (SCH23390 and sulpiride, respectively) or their respective carriers, saline and DMSO, were administered in different doses (0.25, 1, and 4 µg/0.5 µl per rat) into the CA1 area. The analgesic effects of RS were examined with and without dopaminergic receptor antagonists using the tail-flick test. This study's findings indicated that the administration of SCH23390 and sulpiride into the CA1 area of the hippocampus at the maximal doses (4 µg/0.5 µl; *P* < 0.001) markedly diminished the analgesic benefits of RS in the acute pain paradigm. The magnitude of estimated ED50s in the effect of these antagonists on reducing the RS-induced analgesia was 1.64 µg for SCH23390 and 2.79 µg for sulpiride, suggesting that both dopamine D1-like and D2-like receptors in the CA1 region of the hippocampus likely contribute to the analgesic effects of RS in the rats.

## 1 Introduction

Although the perception of acute pain in response to noxious stimuli is an unpleasant experience, it informs the organism of potential tissue damage and triggers behaviors aimed at minimizing injury (Sorkin and Wallace, 1999). Various internal and external factors can affect pain perception, including stress (Macintyre and Schug, 2021). Stress can suppress pain pathways and reduce pain perception, a phenomenon called stress-induced analgesia (SIA) (Butler and Finn, 2009a). SIA can be crucial for an organism's survival because it enables it to suppress pain sensations in high-risk and stressful situations. The mechanisms of SIA are not yet fully understood and depend on the nature of the stress, the intensity, and the duration of exposure to the stressor (Jennings et al., 2014). Chronic stress, unlike short-term stress, may not only not suppress pain but even increase pain responses (Olango and Finn.). Studies show that SIA can be caused by opioid and non-opioid systems (Grisel et al., 1993). The restraint stress (RS) is generally considered to be a psychological stressor that causes significant analgesia through the activation of opioid pathways (Calcagnetti et al., 1990, Dayas et al., 2001).

The hippocampus is one of the brain structures that can be involved in the mechanisms of SIA (Ghasemzadeh and Rezayof, 2015, Merdasi et al., 2022). Although this structure is best known for its role in memory and learning, in recent years, its involvement in processing information related to pain and stress has also been proven (Wingenfeld and Wolf, 2014, Vachon-Presseau et al., 2013). In particular, the CA1 region of the hippocampus is one of the main areas processing received information and relaying it to areas involved in pain and stress pathways, such as the amygdala and the cerebral cortex. Various neurotransmitter systems are involved in information processing in the hippocampus, including the dopaminergic system (Ishikawa et al., 1982). The hippocampus, including the CA1 region, receives dopaminergic projections from the VTA and expresses both types of dopaminergic receptors, namely D1-like receptors (D1R) and D2-like receptors (D2R) (Tsetsenis et al., 2023, Holly and Miczek, 2016).

The distribution pattern of these two receptors is slightly different. D1Rs are typically present in the dentate gyrus and on pyramidal neurons in areas CA1 and CA3. Evidence suggests that these receptors are mainly located postsynaptically (Sotres-Bayón et al., 2001, Harbuz and Lightman, 1989). In contrast, D2Rs, although present in areas CA1 and CA3, are mainly located presynaptically on GABAergic neurons (Bodnar et al., 1978). New studies suggest that dopaminergic signaling in the hippocampus may play a role in regulating pain-related behaviors (Kelly and Franklin, 1987, Butler and Finn, 2009b).

Interestingly, acute stress causes firing of dopaminergic neurons in the VTA (Long et al., 2016). On the other hand, stimulation of the VTA region can cause analgesia (Gamaro et al., 1998). Therefore, the present study was designed to investigate the role of dopaminergic receptors in the CA1 region of the hippocampus in producing the analgesic effects of restraint stress.

Notably, another study has raised a similar question, but in the previous study, forced swim stress (FSS) was used instead of RS as a stress induction model (Ferdousi and Finn, 2018). FSS and RS have different natures. Classifying FSS as either psychological or physical stress is challenging, and it is often regarded as a combination of both types of stress (Ziv et al., 2010). FSS activates non-opioid pathways in its analgesic effects and is not sensitive to the effects of naloxone as an opioid receptor antagonist (Vasic and Schmidt, 2017). In contrast, RS is considered a psychological stressor and activates opioid-dependent analgesic pathways (Ziv et al., 2010, Pani et al., 2000). Based on these distinct mechanisms, we hypothesized that CA1 dopaminergic receptors may contribute differently to analgesia depending on the type of stressor applied. Therefore, by choosing RS as a stress model, the present study aimed to determine the role of dopaminergic receptors in the analgesic effects of restraint stress, an SIA associated with opioid pathways. The answer to this question could help identify the pathways involved in the interaction between stress and pain. Finding these pathways will transform pain management from a simple "block the pain signal" approach to a comprehensive, intelligent approach considering the patient's brain, emotions, and stress. This knowledge paves the way for more effective, safe, and personalized treatments.

## 2 Materials and methods

### 2.1 Animal

The study included ninety-six male Wistar rats, weighing between 200 and 250 g, from the Pasteur Institute in Tehran, Iran. Eleven of these animals were excluded from the study due to incorrect cannula placement. The animals were housed in cages with three per cage under conventional laboratory settings of 22 ◦C and a 12-hour light/12-hour dark cycle. They had unlimited access to food and water. The Research and Ethics Committee, the National Institute for Medical Research Development, approved all investigations and procedures (IR.NIMAD.REC.1404.039) in compliance with the National Institutes of Health Guide, 8th edition, revised 2011.

### 2.2 Drugs

This study utilized these drugs: SCH23390 (Tocris Bioscience, Bristol, UK) was dissolved in saline to act as an antagonist of the D1R. Sulpiride (Tocris Bioscience, Bristol, UK) was diluted in 12 % DMSO to act as a D2R antagonist. Animals under control received 12 % DMSO and saline as a vehicle.

### 2.3 Stereotaxic surgery

Rats were placed into a stereotaxic apparatus (Stoelting, Wood Dale, Illinois, USA) after being given intraperitoneal injections of xylazine (10 mg/kg) and ketamine (100 mg/kg) to induce anesthesia. A longitudinal cut was performed along the centerline, the skin covering the head was detached, and the region surrounding the bregma was sterilized and dried. The stainless steel guide cannula was unilaterally placed into the CA1 region of the hippocampus. The coordinates for this region were determined using the rat brain atlas, with the following measurements (Paxinos and Watson, 2007): Anterior-Posterior = 3–3.5 mm caudal to bregma, Lat = ±1.8 mm lateral to midline, Dorsal-Ventral = 2.8–3 mm ventral from the skull surface (using 23-gauge cannula, 12 mm in length, with the guide cannula positioned 1 mm above the appropriate injection site). Dental acrylic cement was utilized to secure the guiding cannula in the location. The guiding cannula was covered with two stainless steel stylets to avoid the blockade of the cannula by dust during the recovery period. Following the surgical procedure, the animals were placed in separate housing units and given one week to recuperate before commencing the tests.

### 2.4 Drug administration

The microinjections were performed by inserting a 30-gauge stainless steel injector cannula 1 mm longer than the guide cannula into the CA1. The injector cannula was linked to a 1-μL Hamilton syringe using polyethylene tubing (PE-20). Subsequently, a drug solution or vehicle (DMSO or saline) was injected for 60 s and left for an additional 60 s. Following the injection, the covers were repositioned onto the guide cannula. In separate groups of animals, various concentrations of D1R (SCH23390) and D1R (sulpiride) antagonists were delivered gradually into the CA1 over 60 s, with a total volume of 0.5 μL. Each drug solution was made on the day of the test, and all microinjections were administered on both sides.

### 2.5 Behavioral tests

#### 2.5.1 Restraint stress

The animals were relocated to the experimental room and given thirty minutes to adjust. Five minutes after either medication or vehicle microinjection, rats were placed in a restrainer for three hours. The rats were subjected to RS by being placed within Plexiglas tubes (25 cm in length × 6 cm in height) modified with a piston to restrict rat movement. The trials were conducted daily from 8:00 a.m.–4:00 p.m.

#### 2.5.2 Tail-flick test

The tail-flick test was evaluated at 5, 15, 30, 45, and 60 min after intra-CA1 microinjection of drugs (SCH23390 or sulpiride) or vehicles (Saline or 12 % DMSO). The tail-flick apparatus (Harvard Apparatus, USA) determined the nociceptive threshold using the D′amour and Smith method. After 30 min of adaptation, heat was delivered to the upper surface of the rat tails using radiant energy. The heat was administered at distances of 3, 5, and 7 cm from the tail tip. An automatic sensor then measured the animals' tail withdrawal time and recorded it as tail-flick latency (TFL). The mean of the acquired data from three consecutive TFL tests was measured and documented at each time point. The radiant heat source was adjusted to 3–4 s at an intensity level that produces baseline TFL values corresponding to approximately 45 % of the maximum light intensity. A preprogrammed automated cut-off period of 10 s was selected to limit potential tissue harm in cases of no withdrawal response. The following formula was used to express TFL results as a percentage of maximal possible effect (%MPE):

**%MPE** = [Post-drug TFL (s) – Baseline latency (s)] / [Cut-off point value (s) – Baseline latency (s)] * 100

#### 2.5.3 Locomotor activity

Considering that changes in Locomotor activity can affect the results of the tail-flick test, the Locomotor activity of animals in intact, sham, vehicle, SCH23390 (4 µg), and sulpiride (4 µg) was investigated. Following microinjection, rats were placed in an open field (60 × 60 cm2). They were given 10 min to freely explore the arena. A 3CCD camera (Panasonic Inc., Japan) positioned two meters above the open field documented the traveled distance. The data was analyzed offline using Ethovision video tracking software (Version 3.1, Noldus Information Technology, the Netherlands).

### 2.6 Experimental design

**Fig. 1:**
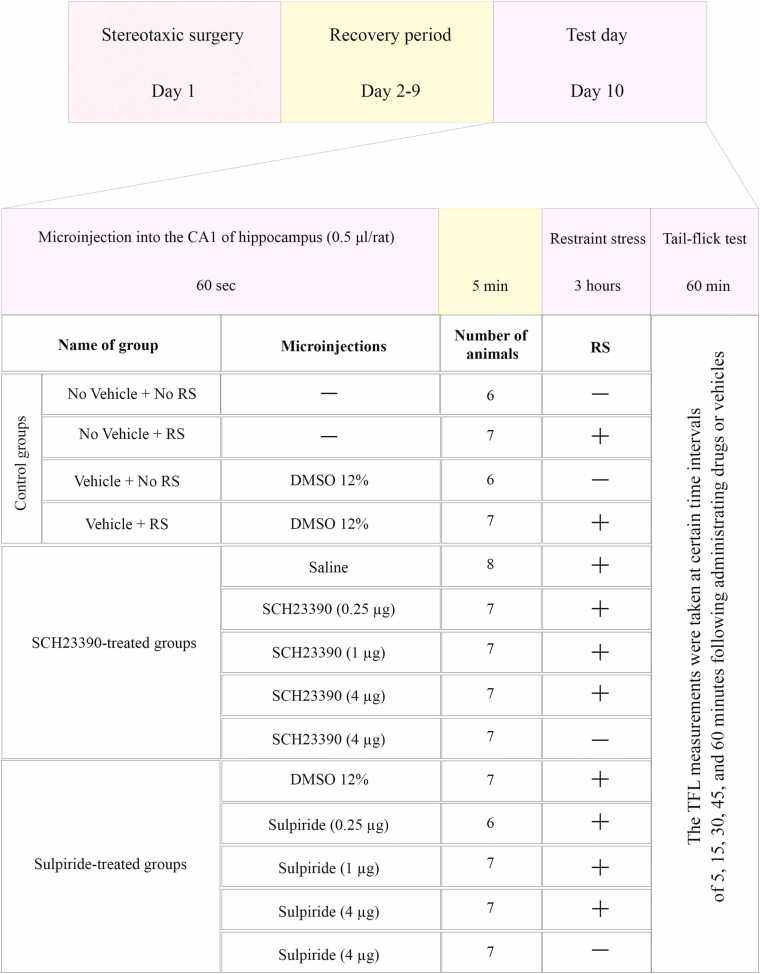
A schematic representation of the experimental protocol for investigating the effect of intra-CA1 injection of SCH23390, a D1-like dopamine receptor antagonist, or sulpiride, a D2-like dopamine receptor antagonist on restraint stress-induced antinociceptive responses.

This study includes three main supergroups of experiments (Fig. 1):

I. *Control groups* were designed to investigate the effects of surgery and vehicle injection on analgesia induced by RS and included the following four subgroups:

• No Vehicle + No RS: The animals in this group underwent surgery and had a cannula implanted in the CA1 region, but they did not receive any stress or microinjections (n = 6).
• No vehicle + RS: The subjects were exposed to 3 h of RS without receiving any microinjections (n = 7).
• Vehicle + No RS: Animals received 0.5 µl of vehicle without experiencing RS (n = 6).
• Vehicle + RS: Animals received a 0.5 µl vehicle microinjection and a 3-hour RS (n = 7).

I. *D1R blockade groups* were designed to characterize the role of hippocampal D1Rs in RS-induced analgesia.

• Dose-response: In three separate subgroups, five minutes before the animals were exposed to RS, different doses of SCH23390 (0.25, 1, or 4 µg/0.5 µl) were injected unilaterally into the hippocampal CA1 region (n = 7).
• Vehicle group: Animals in this group received saline (0.5 µl) as a vehicle 5 min before exposure to RS (n = 7).
• Drug-only control: To determine the effect of SCH23390 alone on acute nociceptive response latency, this group received four µg of SCH23390 without exposure to RS (n = 7).

I. *D2R blockade groups* were used to examine the role of hippocampal D2Rs in mediating the RS-induced analgesia.

• Dose-response: Five minutes before the animals were exposed to RS, different doses of sulpiride (0.25, 1, or 4 µg/0.5 µl) were administered unilaterally into the hippocampal CA1 region in three distinct subgroups (n = 6–7).
• Vehicle group: Animals in this group received 12 % DMSO (0.5 µl) as a vehicle 5 min before exposure to RS (n = 7).
• Drug-only control: This group was administered four µg of sulpiride alone to assess its impact on acute nociceptive response latency, without exposure to RS (n = 7).

### 2.7 Histology

**Fig. 2:**
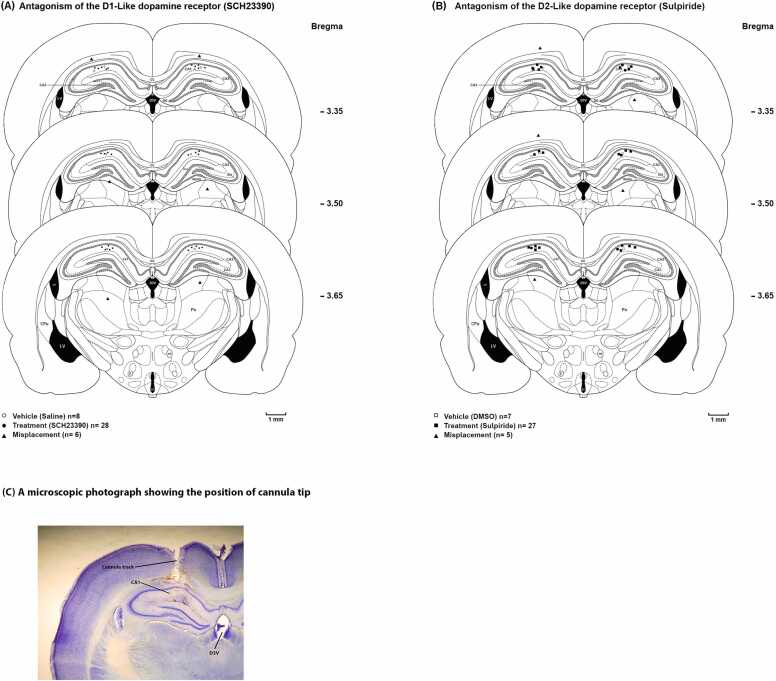
The diagram illustrates the location of unilateral (left or right side of skull midline) microinjection sites in the CA1 region in rat brain coronal sections. **(A)** Microinjection locations for either the vehicle (saline) or D1-like dopamine receptor antagonists (SCH23390). **(B)** Microinjection locations for either the vehicle (12 % DMSO) or D2-like dopamine receptor antagonists (Sulpiride). cc, corpus callosum; CPu, caudate putamen; D3V, dorsal 3rd ventricle; DG, dentate gyrus; DMSO, dimethyl sulfoxide; LV, lateral ventricle. **(C)** A microscopic photograph showing the position of the cannula tip.

After ketamine and xylazine deep anesthesia at the end of each experiment, the animals were transcardially perfused with a solution containing 0.9 % saline and 10 % formalin. Their brains were removed, blocked, and sectioned coronally through the cannula placements in 50-μm sections (Fig. 2). The Paxinos and Watson (2007) rat brain atlas was used to confirm the neuroanatomical location of the cannula tips. Only the animals with correct cannula placements were included in the data analysis.

### 2.8 Statistics

Data were presented as mean ± SEM (standard error of the mean). *P* values less than 0.05 were considered to be statistically significant. The MPE (%) values of tail flicking time in all time set intervals were analyzed using repeated measures (RM) of two-way analysis of variance (ANOVA) followed by the Bonferroni post-*hoc* test. The repeated measures of one-way ANOVA, followed by Dunnett's multiple comparison tests and the student unpaired *t*-test, were used to compare the calculated area under the curve (AUC) obtained from MPE (%) values. The estimated effective dosage of 50 % (ED50) values was computed quantitatively using three different doses of SCH23390 (0.25, 1, and 4 μg/0.5 μl saline) and sulpiride (0.25, 1, and 4 μg/0.5 μl 12 % DMSO) microinjected into the CA1. Using the trend line equation feature in the Excel software (Version 2019), the best-fitted line to represent the scatter plot data was created to evaluate the projected ED50.

## 3 Results

### 3.1 The stress-induced analgesia induced by restraint stress in the No vehicle + RS and Vehicle + RS groups

**Fig. 3:**
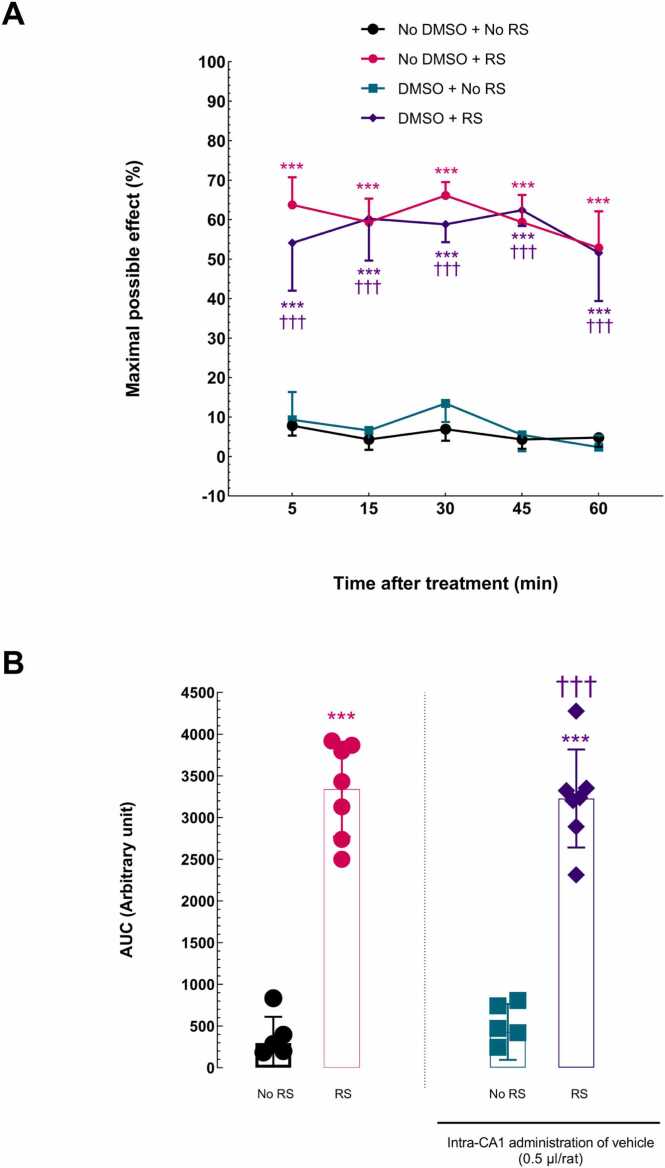
Restraint stress (RS) effect on acute pain-related behaviors. **(A)** A two-way ANOVA followed by the Bonferroni post-*hoc* test was used to compare the percentage of maximal possible effect (MPE%). The tail-flick latency times were taken at 5, 15, 30, 45, and 60 min. **(B)** AUC for MPE% values was compared using ordinary one-way ANOVA followed by the Dunnett post-*hoc* test. For each group of rats, each point displays the mean + SEM. ^***^P < 0.001 compared to the No DMSO + No RS group. ^†††^P < 0.001 compared to the DMSO + No RS group. DMSO, dimethyl sulfoxide.

In this group of experiments, the effect of exposure to RS on acute nociceptive response latency was examined at several intervals (5, 15, 30, 45, and 60 min after RS exposure). The possible effects of vehicle injection or surgery on impact of RS were also evaluated. The calculated % MPEs were assessed using the RM two-way ANOVA with Bonferroni post-*hoc* test. The analysis revealed that RS significantly raised the nociceptive response latency for 60 min after exposure, in the No DMSO + RS and DMSO + RS groups, compared to the No DMSO + No RS group. Furthermore, the acute nociceptive response latency in the DMSO + RS group compared to the DMSO + No RS group significantly increased, indicating that vehicle injection had no effect on analgesia induced by RS [treatment effect: *F* (3, 88) = 55.21, *P* < 0.0001; time effect: *F* (4, 88) = 1.006, *P* = 0.4090, NS; treatment and time interaction effect: *F* (12, 88) = 0.9932, NS; Fig. 3A].

The AUCs for the %MPE data were compared to further measure the overall analgesic effect using an unpaired Student's t-test. The results showed that the acute nociceptive response latency in the No DMSO + RS [t (9) = 12.15, P < 0.0001; Fig. 3B] and DMSO + RS [t (9) = 11.42, P < 0.0001; Fig. 3B] groups increased significantly compared to the No DMSO + No RS group. Moreover, a Student's *t*-test showed that the acute nociceptive response latency in the DMSO + RS group was significantly higher than in the DMSO + No RS group, meaning that injection of vehicle into CA1 had no effect on the analgesic effects of RS [*t*_(11)_ = 10.27, *P* < 0.0001; Fig. 3B].

### 3.2 Effects of microinjection of different doses of D1-like dopamine receptor antagonist into the hippocampal CA1 area on analgesia induced by the restraint stress in an acute pain model

**Fig. 4:**
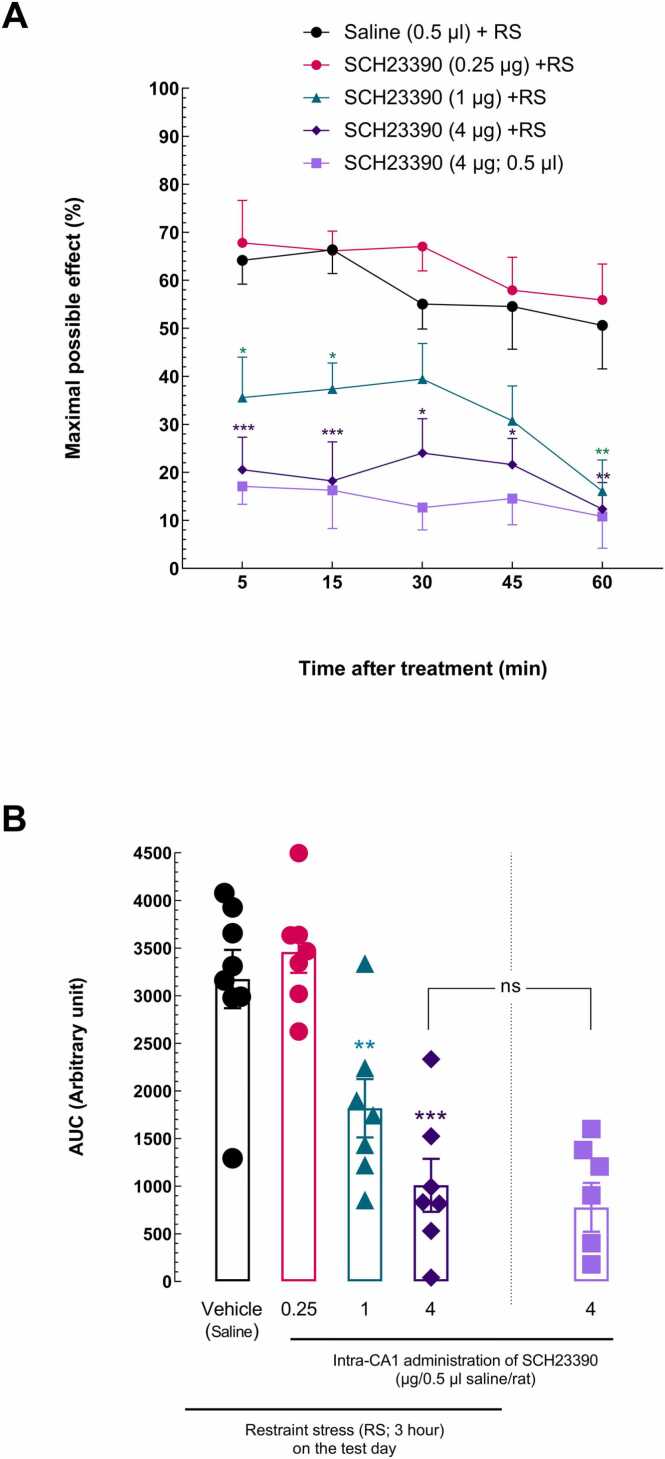
The effect of intra-CA1 injection of SCH23390, a D1-like dopamine receptor antagonist, on restraint stress-induced analgesia. **(A)** A two-way ANOVA followed by the Bonferroni post-*hoc* test was used to compare the MPE% between groups that received different dosages of SCH23390 (0.25, 1, and 4 µg) and the group that received the vehicle (saline) in the CA1 before RS exposure. The tail-flick latency times were taken at 5, 15, 30, 45, and 60 min. **(B)** AUC for MPE% values was compared using ordinary one-way ANOVA followed by the Dunnett post-*hoc* test. For each group of rats, each point displays the mean + SEM. *P < 0.05,^**^P < 0.01^,^ and ^***^P < 0.001 in comparison to the saline + RS group.

The doses of the SCH23390 (0.25, 1, and 4 μg/0.5 μl) as the D1-like dopamine receptor antagonist were administered before stress exposure to examine the impact of D1R in the CA1 on RS-induced analgesia. The results of an RM two-way ANOVA with a Bonferroni post-*hoc* test showed that in the groups receiving 1 and 4 μg/0.5 μl SCH23390, the acute nociceptive response latency was significantly reduced compared to the group receiving saline, indicating the attenuating effect of D1Rs inhibition in the CA1 on analgesia induced by RS [treatment effect: *F* (4, 128) = 20.89, *P* < 0.0001; time effect: *F* (4, 128) = 4.751, *P* = 0.0013; treatment and time interaction effect: *F* (16, 128) = 0.6201, NS; Fig. 4A].

In addition, comparing the AUC using ordinary one-way ANOVA followed by the Dunnett multiple comparisons test confirmed the attenuating effect of D1R antagonist microinjection on analgesia induced by RS [*F* (3,28) = 16.44, *P* < 0.0001; effect size, *η*2 = 0.66; Fig. 4B]. A comparison of the AUCs between the group that received 4 μg/0.5 μl SCH23390 + RS and the group that received 4 μg/0.5 μl SCH23390 alone using a *t*-test showed no significant difference in the acute nociceptive response latency, meaning that SCH23390 microinjection by itself did not affect the acute nociceptive response latency [*t*_(12)_ = 0.6132, *P* = 0.5512; Fig. 4B].

### 3.3 Effects of microinjection of different doses of D2-like dopamine receptor antagonist into the hippocampal CA1 area on analgesia induced by the restraint stress in an acute pain model

**Fig. 5:**
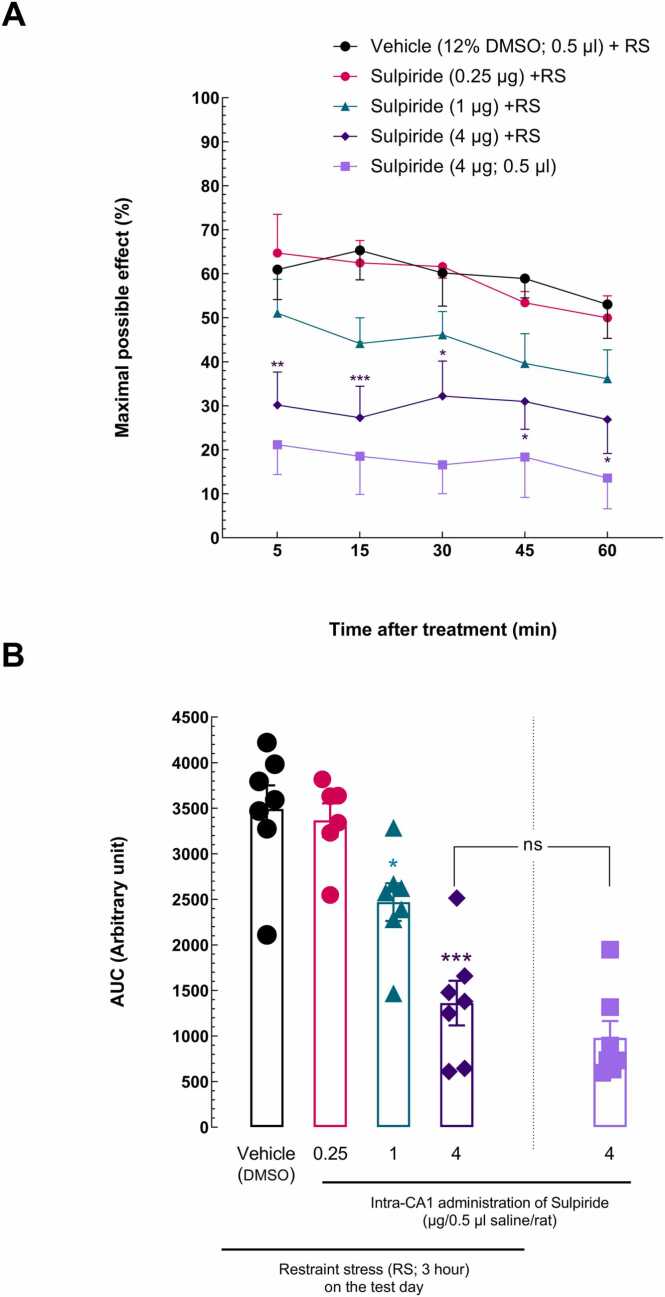
The effect of intra-CA1 injection of sulpiride, a D2-like dopamine receptor antagonist, on restraint stress-induced analgesia. (A) A two-way ANOVA followed by the Bonferroni post-*hoc* test was used to compare the MPE% between groups that received different dosages of sulpiride (0.25, 1, and 4 µg) and the group that received the vehicle (12 % DMSO) in the CA1 before RS exposure. The tail-flick latency times were taken at 5, 15, 30, 45, and 60 min. (B) AUC for MPE% values was compared using ordinary one-way ANOVA followed by the Dunnett post-*hoc* test. For each group of rats, each point displays the mean + SEM. *P < 0.05, ^**^P < 0.01^,^ and ^***^P < 0.001 in comparison to the vehicle (12 % DMSO) + RS group.

The effects of D2R in the CA1 on RS-induced analgesia were investigated by administering the sulpiride doses (0.25, 1, and 4 μg/0.5 μl), as the D2-like dopamine receptor antagonist, prior to RS exposure. The comparison results of a RM two-way ANOVA with a Bonferroni post-*hoc* test indicated that the groups administered 1 and 4 μg/0.5 μl sulpiride + RS exhibited a significant reduction in acute nociceptive response latency relative to the vehicle (12 % DMSO) + RS group, demonstrating the inhibitory effect of D2R antagonism in the CA1 region on RS-induced analgesia [treatment effect: *F* (4, 132) = 9.681, *P* < 0.0001; time effect: *F* (4, 132) = 3.601, *P* = 0.0081; treatment and time interaction effect: *F* (16, 132) = 0.4698, NS; Fig. 5A].

Furthermore, the attenuating impact of D2R antagonist microinjection in the CA1 on analgesia generated by RS was validated by comparing the AUC using an ordinary one-way ANOVA and then the Dunnett multiple comparisons test [*F* (3,26) = 18.67, *P* < 0.0001; effect size, η2 = 0.71 Fig. 5B]. Lastly, the sulpiride has no intrinsic effect on nociception at a dose of 4 μg, according to a *t*-test that revealed no significant difference between animals receiving 4 μg of sulpiride alone and those receiving the same dose prior to RS [*t*_(12)_ = 1.246, P = 0.2365] (Fig. 5B).

### 3.4 Effects of microinjection of D2-like and D1-like dopamine receptor antagonists alone into the hippocampal CA1 area on nociceptive response latency

**Fig. 6:**
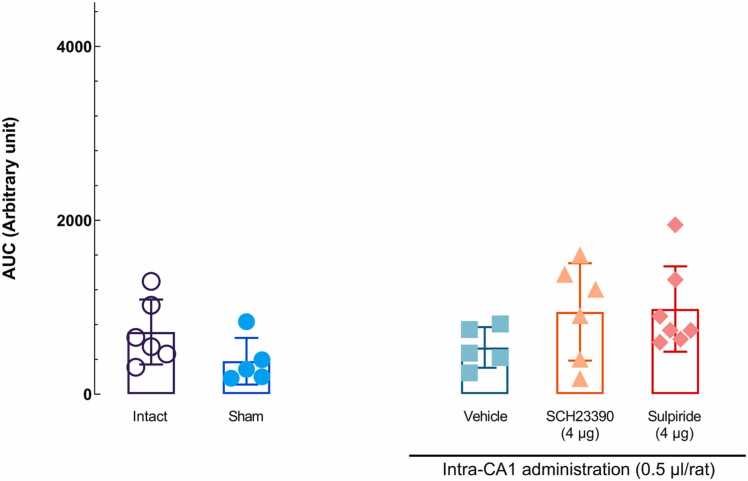
The effect of microinjections of vehicle, sulpiride, and SCH23390 in the CA1 on the tail-flick latency. An ordinary one-way ANOVA followed by Tukey's post-hoc test revealed no significant difference in the AUC for MPE% values between different groups. Each point represents the mean ± SEM.

To investigate the effect of D1R and D2R antagonists on nociceptive response latency in the absence of stress, maximum dose sulpiride (4 μg) and SCH23390 (4 μg) were injected the into the CA1. The comparison results of an ordinary one-way ANOVA and then the Dunnett multiple comparisons test indicated that the sulpiride and SCH23390 have no intrinsic effect on tail-flick latency at a dose of 4 μg [*F* (4,24) = 2.154, *P* = 0.1051; Fig. 6].

### 3.5 Comparison of the calculated ED50 values of the intra-CA1 microinjection of D1- and D2-like dopamine receptor antagonists on the restraint stress-induced analgesia

**Fig. 7:**
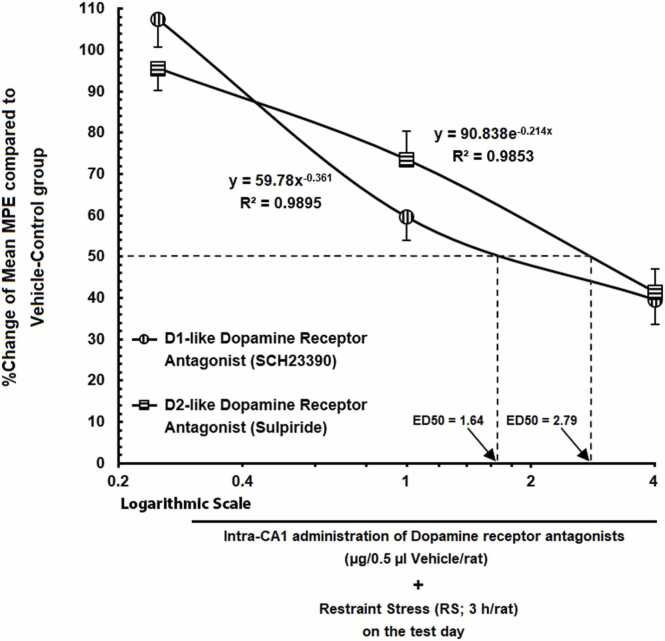
A log dose-response curve showing how SIA is affected by intra-CA1 microinjection of varying dosages of SCH23390 or sulpiride (0.25, 1, and 4 μg). Sulpiride had a 50 % effective dosage (ED50) of 2.79, which was higher than SCH23390's (1.64).

The estimated ED50 was calculated to be 1.64 μg for SCH23390 and 2.79 μg for sulpiride. These values were derived from the best-fit line on a log dose-response curve (Fig. 7), plotted using Excel software. These findings indicate that D1R in the CA1 plays a more prominent role in mediating the RS-induced analgesia in this animal model of pain.

### 3.6 Comparison of the effects of surgery, vehicle microinjection, and blocking the D1R and D2R in the hippocampal CA1 area on locomotor activity

**Fig. 8:**
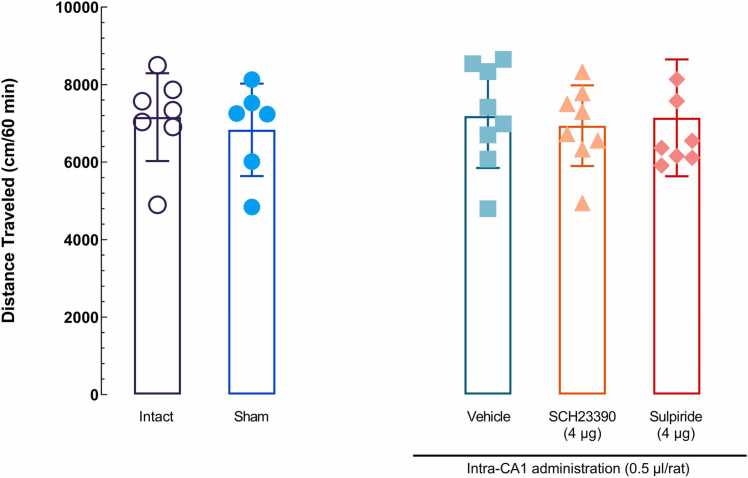
The effect of microinjections of vehicle, sulpiride, and SCH23390 in the CA1 on the motor activity of rats. An ordinary one-way ANOVA followed by the Tukey's post-*hoc* test revealed no significant difference in the distance traveled during the open field test between different groups. Each point represents the mean ± SEM.

A one-way ANOVA, accompanied by Tukey's multiple comparison tests, indicated that neither surgery, vehicle administration, nor the effective doses of SCH23390 and sulpiride (4 μg/0.5 μl per rat) administered into the CA1 influenced locomotor activity [*F* (4, 32) = 0.1070, *P* = 0.9792; Fig. 8].

## 4 Discussion

Current research demonstrates the role of dopaminergic receptors in the CA1 area of the hippocampus in SIA resulting from RS. Results indicate that (i) exposure to RS for 3 h can increase the threshold of acute pain responses and produce analgesia. In contrast, (ii) administering D1R (SCH23390) and D2R (sulpiride) antagonists intra-CA1 microinjection prior to RS significantly diminishes the analgesic impact of this kind of stress. However, (iii) microinjection of SCH23390 and sulpiride alone in this area has no effect on the threshold of acute pain responses. These findings indicate that the dopaminergic pathway in the CA1 region of the hippocampus is a critical component in modulating pain under acute stress. The SIA is an adaptive and survival response in which an organism under threatening conditions shows a reduced response to painful stimuli (Butler and Finn, 2009b). However, evidence suggests that stress has a dual effect on pain. That is, depending on the nature, intensity, and duration of exposure to stress, its effect on pain can be an inhibitory or facilitatory action (Jennings et al., 2014). SIA is usually induced when the stressor is severe and acute. A diverse set of psychological and physical stresses can induce SIA in different pain models. Several studies have examined the effect of exposure to RS as a psychological stressor on acute pain responses, and the results of these studies indicate that acute exposure to RS can increase the threshold for pain responses (Long et al., 2016, Gamaro et al., 1998).

Many efforts have been made to understand the mechanisms involved in developing SIA. While the role of opioid systems and descending pain inhibitory pathways in this phenomenon is well known (Ferdousi and Finn, 2018), the role of non-opioid systems, such as the dopaminergic system, has been increasingly recognized. The hippocampus, classically associated with learning and memory, is also recognized as an essential center for processing the emotional information of pain (Ziv et al., 2010, Vasic and Schmidt, 2017). The obtained results strengthen the view that the hippocampus is involved not only in the cognitive and emotional aspects of pain but also in the direct modulation of its intensity. The mesolimbic dopaminergic system, originating in the ventral tegmental area (VTA) and innervating structures such as the hippocampus, amygdala, and nucleus accumbens, is highly activated in response to stressful stimuli (Pani et al., 2000, Baik, 2020).

As used in this study, the RS is a potent agent for increasing dopamine release (Carlson et al., 1991). The present findings suggest that this stress-induced dopamine increase functions beyond the modulation of stressful memory and is directly involved in generating an analgesic state. This is consistent with previous studies indicating a role for the dopaminergic system in other brain regions in SIA. Blocking dopaminergic receptors in the nucleus accumbens, VTA, and dentate gyrus (DG) can reduce the analgesic effects of stress in models of acute and inflammatory pain (Faramarzi et al., 2016, Golmohammadi et al., 2024, Saghafi et al., 2025). In addition, our previous study investigated the role of CA1 dopaminergic receptors in FSS-induced SIA (Shirmohammadi et al., 2025). This study's results were also consistent with the results of the present study. FSS and RS are two different types of stress and may activate different pathways in brain circuits (Neumann et al., 1998). FSS is a mixture of both physical and psychological stress that produces most of its analgesic effects through non-opioid pathways (Bodnar et al., 1978, Mogil et al., 1996). While RS is generally considered a psychological stress, the involvement of the opioid system in its analgesic effects has been proven (Grisel et al., 1993, Calcagnetti et al., 1990). The results of these two studies may indicate the key role of hippocampal dopaminergic pathways in producing the analgesic effects of different types of stress.

One of the intriguing findings of this study is the concurrent role of both D1 and D2 dopamine receptor families. These two receptor families often have opposing effects on cellular activity; D1Rs are typically excitatory and facilitate synaptic plasticity by increasing cAMP, whereas D2Rs are generally inhibitory and decrease cAMP levels (Neve et al., 2004). The fact that blockade of either receptor alone reduces SIA suggests a complex regulatory mechanism. A precise balance between D1 and D2 signaling in CA1 neuronal circuits may be necessary to elicit an entire analgesic response. This balance could regulate the membrane potential of CA1 pyramidal neurons in a way that alters the processing of incoming pain signals from the cerebral cortex or thalamus, ultimately affecting hippocampal output to other pain modulation centers such as the amygdala and prefrontal cortex. Notably, the D1R antagonist was effective at lower doses (lower ED50) compared to the D2R antagonist. Although this advantage could be attributed to the specific efficacy and affinity of the pharmacological agents, it might also suggest a greater involvement of dopaminergic excitatory pathways in SIA. However, previous studies that used FSS as a physical stressor instead of RS found that both dopaminergic receptors had the same effects or that D2Rs played a more prominent role (Golmohammadi et al., 2024, Shirmohammadi et al., 2025). These results strengthen the hypothesis that different types of stress activate different pathways despite having the same effects.

The functional connection between the hippocampus and classical pain modulation pathways should be considered. Modern tracing methods show that the dorsal and ventral hippocampus project to the PAG (Wei et al., 2021). Previous studies have extensively demonstrated the role of the PAG as part of descending pain control pathways (Bannister, 2019). Furthermore, the connection between the mesolimbic system and the PAG–RVM descending system is well characterized (Tobaldini et al., 2019). Therefore, it is possible that dopaminergic signaling in hippocampal CA1, by modulating the output of this region to other limbic structures, ultimately alters the activity of PAG neurons and thus contributes to SIA. This hypothesis is an attractive avenue for future research.

This study also has some limitations. Using more specific techniques, such as optogenetics or chemogenetics, to activate or inhibit neurons expressing D1 or D2 receptors in CA1 could significantly increase the understanding of the cellular circuits involved. Direct measurement of dopamine levels in CA1 using microdialysis during stress and pain testing could also directly confirm our findings. Altogether, this study provides strong evidence that the dopaminergic system in the CA1 region of the hippocampus is a key mediator of the acute stress-induced analgesic response. This effect is exerted through the coordinated activation of D1Rs and D2Rs. These findings not only expand the understanding of the neural mechanisms of SIA but also propose the hippocampus as a potential therapeutic target for the management of stress- and pain-related disorders.

## CRediT authorship contribution statement

**Shima Abtin:** Writing – original draft. **Abbas Haghparast:** Writing – review & editing, Supervision, Formal analysis, Conceptualization. **Diba Shirmohammadi:** Investigation, Data curation. **Homayoon Golmohammadi:** Data curation.

## Declaration of Competing Interest

There are no conflicts of interest.

## References

[bib1] Baik J.-H. (2020). Stress and the dopaminergic reward system. Exp. Mol. Med..

[bib2] Bannister K. (2019). Descending pain modulation: influence and impact. Curr. Opin. Physiol..

[bib3] Bodnar R.J., Kelly D.D., Spiaggia A., Pavlides C., Glusman M. (1978). Stress-induced analgesia: Effect of naloxone following cold water swims. Bull. Psychon. Soc..

[bib4] Butler R.K., Finn D.P. (2009). Stress-induced analgesia. Prog. Neurobiol..

[bib5] Butler R.K., Finn D.P. (2009). Stress-induced analgesia. Prog. Neurobiol..

[bib6] Calcagnetti D.J., Fleetwood S.W., Holtzman S.G. (1990). Pharmacological profile of the potentiation of opioid analgesia by restraint stress. Pharmacol. Biochem. Behav..

[bib7] Carlson J.N., Fitzgerald L.W., Keller R.W., Glick S.D. (1991). Side and region dependent changes in dopamine activation with various durations of restraint stress. Brain Res.

[bib8] Dayas C., Buller K., Crane J., Xu Y., Day T.A. (2001). Stressor Categorization: Acute Physical and Psychological Stressors Elicit Distinctive Recruitment Patterns in the Amygdala and in Medullary Noradrenergic Cell Groups. Eur. J. Neurosci..

[bib9] Faramarzi G., Zendehdel M., Haghparast A. (2016). D1-and D2-like dopamine receptors within the nucleus accumbens contribute to stress-induced analgesia in formalin-related pain behaviours in rats. Eur. J. Pain..

[bib10] Ferdousi M., Finn D.P. (2018). Stress-induced modulation of pain: role of the endogenous opioid system. Prog. brain Res..

[bib11] Gamaro G., Xavier M., Denardin J., Pilger J., Ely D., Ferreira M., Dalmaz C. (1998). The effects of acute and repeated restraint stress on the nociceptive response in rats. Physiol. Behav..

[bib12] Ghasemzadeh Z., Rezayof A. (2015). Ventral hippocampal nicotinic acetylcholine receptors mediate stress-induced analgesia in mice. Prog. NeuroPsychopharmacol. Biol. Psychiatry.

[bib13] Golmohammadi H., Shirmohammadi D., Mazaheri S., Haghparast A. (2024). D2-like dopamine receptors blockade within the dentate gyrus shows a greater effect on stress-induced analgesia in the tail-flick test compared to D1-like dopamine receptors. Behav. Pharmacol..

[bib14] Grisel J.E., Fleshner M., Watkins L.R., Maier S.F. (1993). Opioid and nonopioid interactions in two forms of stress-induced analgesia. Pharmacol. Biochem. Behav..

[bib15] Harbuz M.S., Lightman S.L. (1989). Responses of hypothalamic and pituitary mRNA to physical and psychological stress in the rat. J. Endocrinol..

[bib16] Holly E.N., Miczek K.A. (2016). Ventral tegmental area dopamine revisited: effects of acute and repeated stress. Psychopharmacology.

[bib17] Ishikawa K., Ott T., McGaugh J.L. (1982). Evidence for dopamine as a transmitter in dorsal hippocampus. Brain Res..

[bib18] Jennings E.M., Okine B.N., Roche M., Finn D.P. (2014). Stress-induced hyperalgesia. Prog. Neurobiol..

[bib19] Kelly S.J., Franklin K.B.J. (1987). Role of peripheral and central opioid activity in analgesia induced by restraint stress. Life Sci..

[bib20] Long C.C., Sadler K.E., Kolber B.J. (2016). Hormonal and molecular effects of restraint stress on formalin-induced pain-like behavior in male and female mice. Physiol. Behav..

[bib21] Macintyre P.E., Schug S.A. (2021).

[bib22] Merdasi P.G., Dezfouli R.A., Mazaheri S., Haghparast A. (2022). Blocking the dopaminergic receptors in the hippocampal dentate gyrus reduced the stress-induced analgesia in persistent inflammatory pain in the rat. Physiol. Behav..

[bib23] Mogil J.S., Sternberg W.F., Balian H., Liebeskind J.C., Sadowski B. (1996). Opioid and Nonopioid Swim Stress-Induced Analgesia: A Parametric Analysis in Mice. Physiol. Behav..

[bib24] Neumann I., Johnstone H., Hatzinger M., Liebsch G., Shipston M., Russell J., Landgraf R., Douglas A. (1998). Attenuated neuroendocrine responses to emotional and physical stressors in pregnant rats involve adenohypophysial changes. J. Physiol..

[bib25] Neve K.A., Seamans J.K., Trantham-Davidson H. (2004). Dopamine receptor signaling. J. Recept. Signal Transduct..

[bib26] W.M. Olango, D.P. Finn, Neurobiology of stress-induced hyperalgesia, in: Behavioral neurobiology of chronic pain, Springer, pp. 251-280. 10.1007/7854_2014_302 24850075

[bib27] Pani L., Porcella A., Gessa G.L. (2000). The role of stress in the pathophysiology of the dopaminergic system. Mol. Psychiatry.

[bib28] Saghafi M., Danesh E., Ghalandari-Shamami M., Mousavi Z., Haghparast A. (2025). Contribution of D1-and D2-like dopamine receptors in the ventral tegmental area to the stress-induced analgesia in the animal model of acute pain. Behav. Brain Res..

[bib29] Shirmohammadi D., Golmohammadi H., Seyedaghamiri F., Haghparast A. (2025). Role of D1-and D2-like dopamine receptors within the CA1 hippocampal region in the stress-induced antinociceptive response in the exposure to acute pain. Behav. Pharmacol..

[bib30] Sorkin L.S., Wallace M.S. (1999). Acute pain mechanisms. Surg. Clin. North Am..

[bib31] Sotres-Bayón F., Torres-López E., López-Ávila A., Del Ángel R., Pellicer F. (2001). Lesion and electrical stimulation of the ventral tegmental area modify persistent nociceptive behavior in the rat. Brain Res..

[bib32] Tobaldini G., Sardi N.F., Guilhen V.A., Fischer L. (2019). Pain Inhibits Pain: an Ascending-Descending Pain Modulation Pathway Linking Mesolimbic and Classical Descending Mechanisms. Mol. Neurobiol..

[bib33] Tsetsenis T., Broussard J.I., Dani J.A. (2023). Dopaminergic regulation of hippocampal plasticity, learning, and memory. Front. Behav. Neurosci..

[bib34] Vachon-Presseau E., Roy M., Martel M.-O., Caron E., Marin M.-F., Chen J., Albouy G., Plante I., Sullivan M.J., Lupien S.J. (2013). The stress model of chronic pain: evidence from basal cortisol and hippocampal structure and function in humans. Brain.

[bib35] Vasic V., Schmidt M.H. (2017). Resilience and vulnerability to pain and inflammation in the hippocampus. Int. J. Mol. Sci..

[bib36] Wei X., Centeno M.V., Ren W., Borruto A.M., Procissi D., Xu T., Jabakhanji R., Mao Z., Kim H., Li Y., Yang Y., Gutruf P., Rogers J.A., Surmeier D.J., Radulovic J., Liu X., Martina M., Apkarian A.V. (2021). Activation of the dorsal, but not the ventral, hippocampus relieves neuropathic pain in rodents. PAIN.

[bib37] Wingenfeld K., Wolf O.T. (2014). Stress, memory, and the hippocampus. Front Neurol. Neurosci..

[bib38] Ziv M., Tomer R., Defrin R., Hendler T. (2010). Individual sensitivity to pain expectancy is related to differential activation of the hippocampus and amygdala. Hum. brain Mapp..

